# An Unusual Case of Primary Plasma Cell Leukemia Presenting As Pericardial Effusion

**DOI:** 10.7759/cureus.92483

**Published:** 2025-09-16

**Authors:** Ahmed Qaedi, Ethan Barnes, Ali Hachem, Farrah Ibrahim

**Affiliations:** 1 Internal Medicine, University of Alabama at Birmingham, Huntsville Regional Medical Campus, Huntsville, USA; 2 Hematology-Oncology, Southern Cancer Center, Huntsville, USA

**Keywords:** acute pericardial effusion, bone marrow infiltration, immunomodulator therapy, non-secretory plasma cell leukemia, plasma cell disorders

## Abstract

Plasma cell leukemia represents the rarest form of plasma cell dyscrasias arising from monoclonal proliferation of plasma cells in peripheral blood. Its aggressive natural history raises a significant therapeutic challenge. We present a case of a 58-year-old female who presented with a cough accompanied by exertional dyspnea. Further radiological investigations revealed a circumferential pericardial effusion. A pericardiocentesis was performed, resulting in the drainage of 650 cc of hemorrhagic pericardial fluid. Cytology revealed monoclonal plasma cells expressing CD38 and CD138 with cytoplasmic lambda light chain restriction. A bone marrow biopsy disclosed a hypercellular marrow with a monoclonal plasma cell population of 33%. Treatment was initiated with intravenous dexamethasone and weekly subcutaneous injections of bortezomib, followed by outpatient cycles consisting of bortezomib, lenalidomide, daratumumab, and dexamethasone, with a good initial response. However, five months later, the plasma cell leukemia became refractory to treatment, and the patient passed away from septic shock secondary to community-acquired pneumonia. This case illustrates the variable presentations of plasma cell leukemia, as well as its poor prognosis. Further research is warranted to optimize therapeutic regimens and enhance overall survival.

## Introduction

Plasma cell leukemia (PCL) is characterized by peripherally circulating monoclonal plasma cells that can arise de novo (termed primary) or transform into a leukemic phase of previously diagnosed multiple myeloma (termed secondary). It represents an exceptionally aggressive and rare plasma cell dyscrasia with a crude incidence of 0.4 cases per million [1]. The initial definition of PCL was portrayed in 1974, based on the presence of ≥20% plasma cells in peripheral blood with an absolute plasma cell count ≥2 x10^9^/L [2]. The International Myeloma Working Group (IMWG) devised that the initial criteria were too bounded and can lead to false negative diagnoses [3]. In view of the observation that patients with ≥5% circulating plasma cells in peripheral blood had a similar prognosis to those that satisfy the prior definition, IMWG has revised the criteria to a cut-off of ≥5% of plasma cells in peripheral blood to satisfy the diagnosis of PCL [3]. The pathophysiology behind the development of PCL relates to disruption in the cellular pathways responsible for adhering the plasma cells in the bone marrow. Most notably, this includes decreased expression of neural cell adhesion molecule (CD56) and leukocyte function-associated antigen-1 (LFA-1), which secures plasma cells in bone marrow stroma in normal circumstances [4,5]. Furthermore, the leukemic plasma cells have elevated expression of integrin α4β1, which grants their invasive properties and allows the plasma cells to extravasate out of the vascular capillary wall [4,6]. The mortality rate of PCL remains high, which stems from its rapid progression and challenging sensitivity to cytoreductive agents. Herein, we present a case of primary plasma cell leukemia presenting as malignant pericardial effusion.

## Case presentation

A 58-year-old female with a past medical history of trisomy 21, asthma, hypertension, and hypothyroidism presented to the emergency department with a one-day history of shortness of breath, which is aggravated by exertion and relieved on rest. Additionally, she experienced a cough productive of occasional brown sputum. On a background of increasing lower extremity swelling over the past several weeks. She denied any history of orthopnea, fevers, chills, nausea, vomiting, diarrhea, or recent illness or contact with sick individuals. Objectively, the patient had a blood pressure of 147/89 mmHg, a heart rate of 82 bpm, and a respiratory rate of 20 breaths per minute. She was afebrile and maintaining her oxygen saturation at 98% while on 4L of oxygen via nasal cannula. Physical examination was remarkable for bilateral expiratory wheezes on auscultation. She had a regular heart rate and rhythm, with S1 and S2 heart sounds present and no murmurs, gallops, or rubs appreciated. Bilateral lower extremity non-pitting pedal edema was noted.

**Figure 1:**
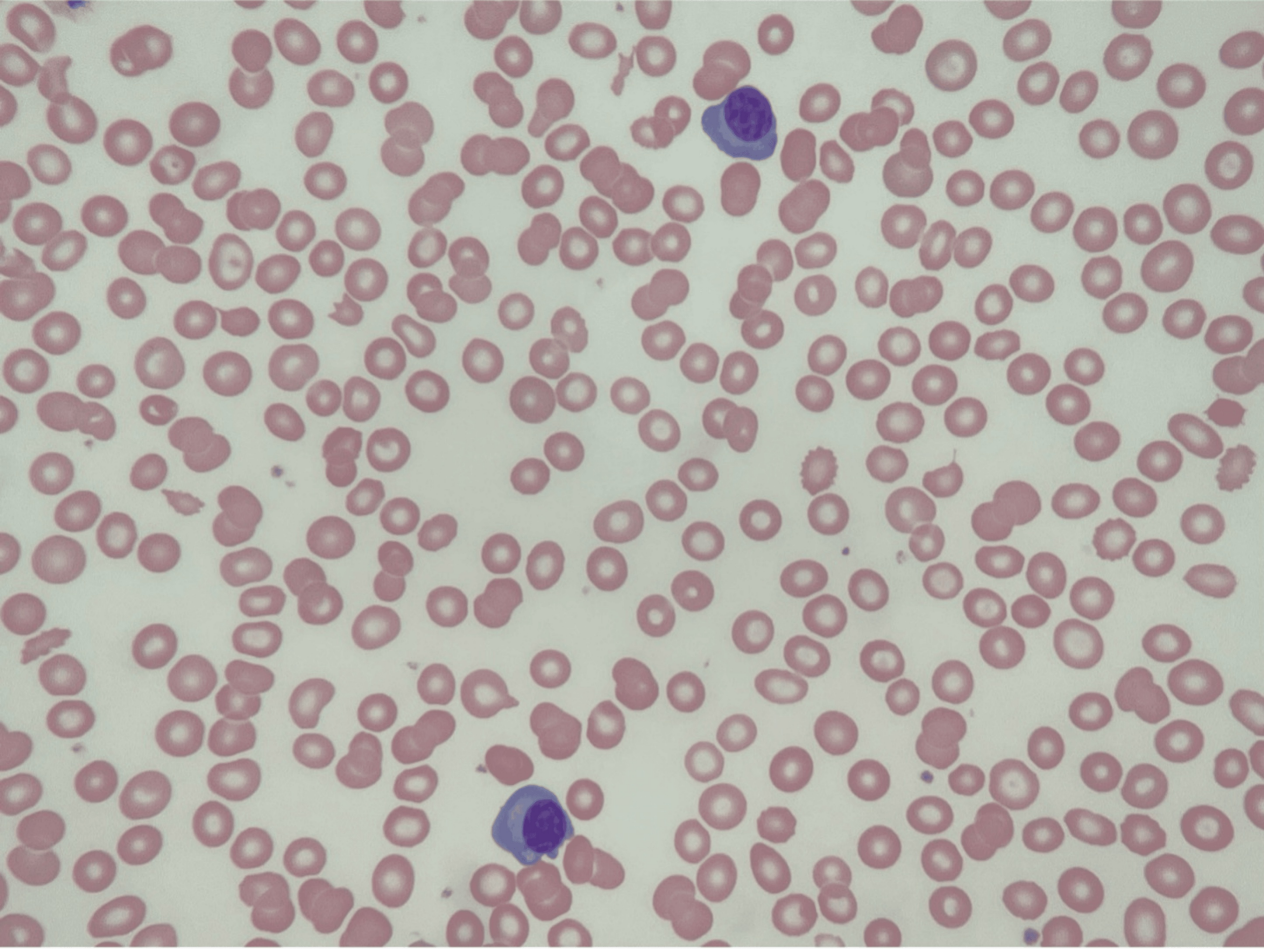
Peripheral blood smear (magnification x40) demonstrating circulating plasma cells.

**Figure 2:**
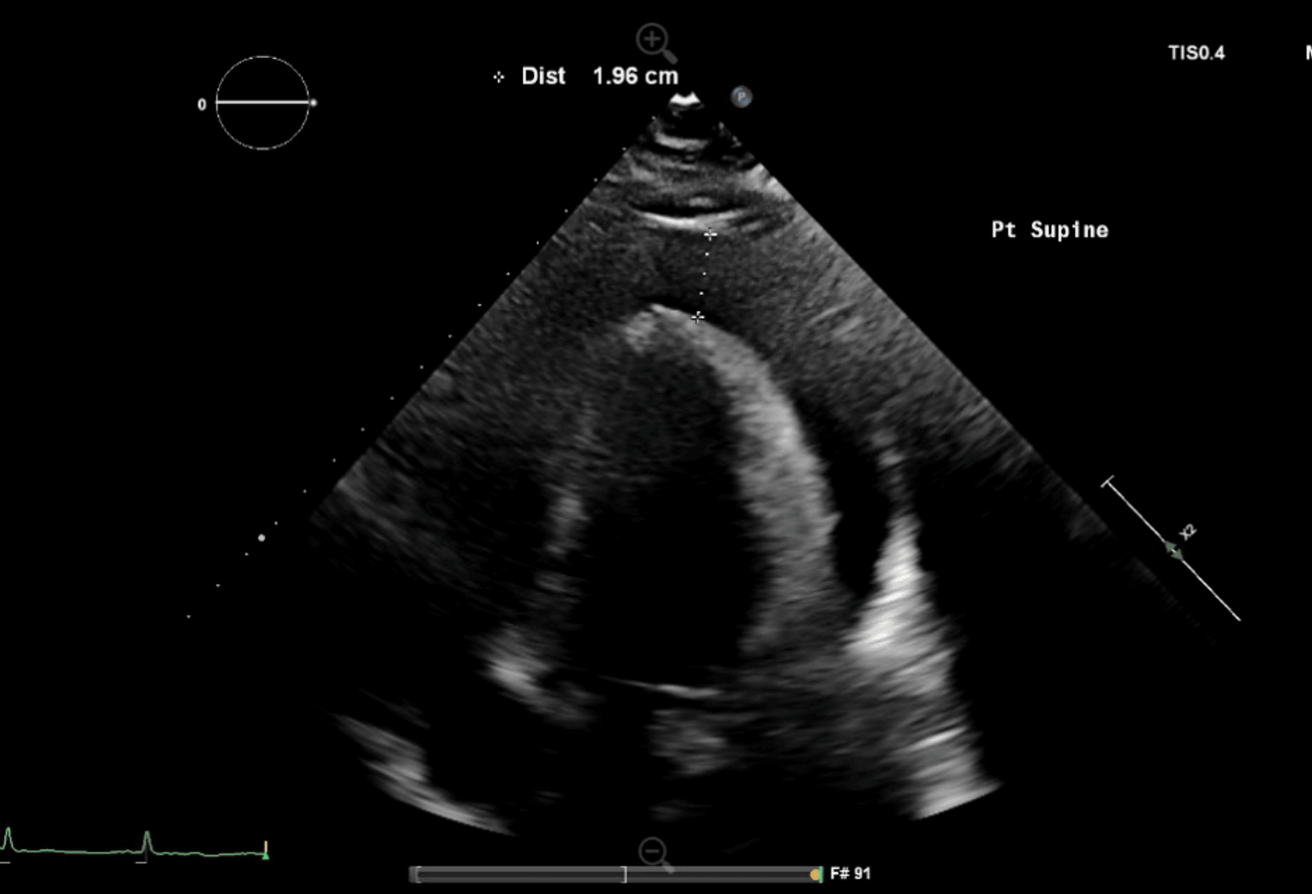
4-chamber view of a transthoracic echocardiogram demonstrating pericardial effusion with a maximum effusion width of 1.96 cm at the end of diastole.

Laboratory investigations (Table 1) revealed elevated serum creatinine along with elevated metamyelocyte and myelocyte levels. Peripheral blood smear revealed circulating plasma cells (Figure 1). Serum protein electrophoresis (Table 2) and urine protein electrophoresis were significant for a monoclonal band of IgG lambda specificity. Peripheral blood flow cytometry demonstrated monoclonal plasma cells expressing plasma cell markers CD38 and CD138, and were negative for CD19, CD20, and CD56. A normal sinus rhythm was evident on the electrocardiogram (EKG), and a chest x-ray revealed interstitial pulmonary edema with bilateral pleural effusions, as well as cardiomegaly reflecting a new pericardial effusion. An echocardiogram illustrated moderate to large pericardial effusion (Figure 2) with normal left ventricular cavity size and function.

**Table 1:** Initial laboratory values demonstrating elevated serum creatinine, myelocyte and metamyelocyte levels. CO2: serum carbon dioxide; BUN: blood urea nitrogen;  LDH: lactate dehydrogenase; Pro-BNP: pro-B-type natriuretic peptide; WBC: white blood cells; Hb: hemoglobin; MCV: mean corpuscular volume.

Laboratory marker	Value	Normal range
Sodium	139 mmol/L	135 – 145 mmol/L
Potassium	4.3 mmol/L	3.5 – 5.0 mmol/L
Chloride	105 mmol/L	96 – 108 mmol/L
CO2	23 mmol/L	22 – 29 mmol/L
BUN	26 mg/dL	6 – 20 mg/dL
Serum Creatinine	1.3 mg/dL (Baseline 0.8)	0.5 – 1.0 mg/dL
Serum Glucose	107 mg/dL	70 – 100 mg/dL
Calcium	9.9 mg/dL	8.6 – 10 mg/dL
Anion Gap	10	7 - 17
Total protein	9.3 g/dL	6.4 – 8.3 g/dL
Albumin	3.3 g/dL	3.5 – 5.2 g/dL
LDH	335 EnzU/L	94 – 250 EnzU/L
Troponin	12 ng/L	<20 ng/L
Pro-BNP	246 pg/L	<242 pg/L
WBC	15.45 x10^3^/microliter	4.5 – 11 x10^3^/microliter
Hb	7.9 g/dL	11.5 – 14.7 g/dL
Platelets	178 x10^3^/microliter	150 – 450 x10^3^/microliter
MCV	93.4 fL	80 – 100 fL
Metamyelocyte	3.6 %	0%
Myelocyte	2.7 %	0%

**Table 2:** Serum protein electrophoresis demonstrating monoclonal IgG band with lambda specificity.

Serum Protein Electrophoresis		
Lab marker	Value	Normal range
Total protein	7.3 g/dL	6.4 – 8.3 g/dL
Albumin	2.64 g/dL	3.57 – 5.49 g/dL
Alpha 1	0.29 g/dL	0.19 – 0.41 g/dL
Alpha 2	0.58 g/dL	0.45 – 0.98 g/dL
Beta	0.52 g/dL	0.54 – 1.09 g/dL
Gamma	3.27 g/dL	0.71 – 1.56 g/dL
Lambda free light chains	96.65 mg/L	5.71-26.3 mg/L
Kappa free light chain	10.8 mg/L	3.3-19.4 mg/L
Kappa to lambda light chain ratio	0.11	0.26-1.65

**Figure 3:**
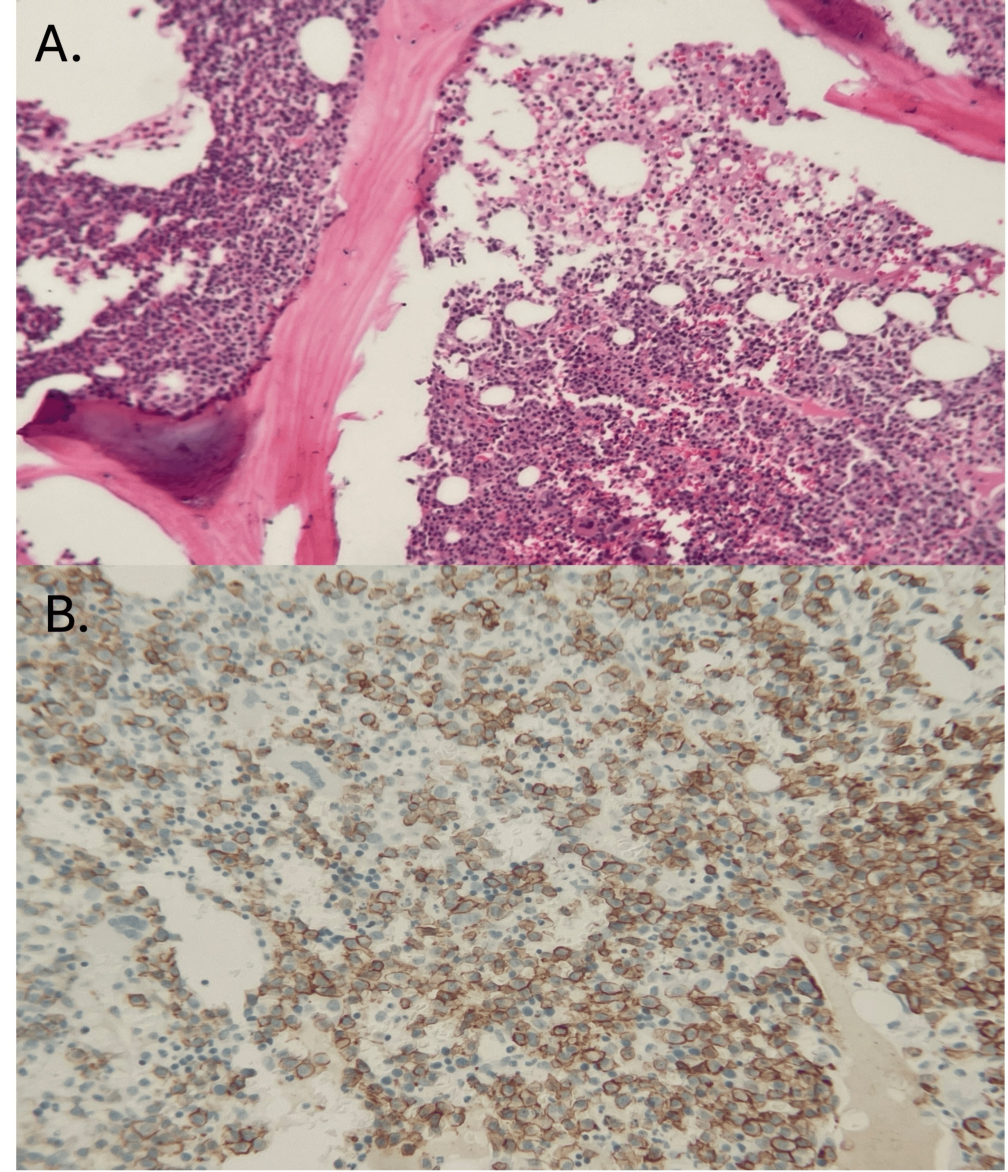
(A) Bone marrow biopsy at low power magnification (x20) demonstrating hypercellularity with abundant appearing plasma cell. (B) CD138 immunohistochemical staining of bone marrow biopsy outlining plasma cells comprising 70% of cellularity.

The patient underwent a pericardiocentesis, which resulted in the drainage of 650 cc of hemorrhagic pericardial fluid with a pericardial catheter drain left in situ. Fluid studies with nucleated cell differential showed neutrophils 27%, lymphocytes 16%, eosinophils 1%, and mononuclear cells 56%. Pericardial fluid cytology showed plasma cells admixed with other mononuclear inflammatory cells, and accompanying flow cytometric analysis revealed a monoclonal plasma cell population 53%, expressing plasma cell markers CD38 and CD138 with cytoplasmic lambda light chain restriction. Fluid cultures remained negative. Following these findings, a bone marrow biopsy confirmed a hypercellular marrow 95% with a plasma cell population of 33%, mild reticulin fibrosis, absent sustainable iron, and negative Congo-red stain for amyloid deposition (Figure 3). The patient received dexamethasone 40mg IV for four days as well as a weekly injection of bortezomib 2.9mg subcutaneously. A repeated echocardiogram showed no clinical evidence of pericardial effusion. Furthermore, the patient was started on acyclovir 400mg twice daily and allopurinol 300mg orally daily for herpes simplex/herpes zoster virus and tumor lysis syndrome prophylaxis, respectively. The patient was discharged and continued her chemotherapy regimen consisting of bortezomib, lenalidomide, daratumumab, and dexamethasone in the outpatient setting with a good initial response. However, 5 months later, her plasma cell leukemia became refractory to treatment, and the patient passed away from acute respiratory failure with septic shock secondary to infectious community-acquired pneumonia.

## Discussion

Plasma cell leukemia represents one of the most aggressive forms of plasma cell dyscrasia. Although new therapeutic modalities are being developed, the prognosis remains poor. A non-curative treatment approach is generally implemented to improve the length and quality of life. Bortezomib-based chemotherapy and accompanying immunomodulatory drugs are currently considered induction therapy for PCL [7]. However, there is no currently standard first-line regimen, and the choice depends on the patient’s goals of care, age, and comorbidities. The most aggressive regimen is bortezomib, dexamethasone, thalidomide, cisplatin, doxorubicin, cyclophosphamide, and etoposide (VDT-PACE) [8]. In addition, bortezomib, lenalidomide, and dexamethasone (VRd) can also be considered alone or in combination with anti-CD38 monoclonal antibody (daratumumab) [9,10]. Furthermore, lanalidomide and/or bortezomib can be continued as maintenance treatments [11]. Certain cytogenetic analyses identified via fluorescent in situ hybridization (FISH) may confer prognostic implications. Our patient possessed the del (13q), which has been associated with a decreased overall survival [12,13].

Hematopoietic stem cell transplantation (HSCT) represents another treatment modality that demonstrated favorable outcomes when compared to non-transplant approaches [14]. Jamison et al. reported a case of pericardial effusion secondary to relapsed multiple myeloma that underwent autologous stem cell transplantation with maintenance therapy consisting of pomalidomide and bortezomib [15]. Notably, no evidence of disease progression was seen in 3 years [15]. Historically median overall survival (OS) of primary PCL ranges from four to 11 months [7]. However, with the introduction of autologous HSCT, there has been an associated increase in median OS to 33 months [14]. Unfortunately, our patient could not proceed with HSCT due to multiple co-morbidities and progression of her PCL. Previously described cases of plasma cell infiltration of the pericardium were associated with similar unsatisfactory outcomes [16,17]. Regarding relapse/refractory primary PCL, salvage therapy can be considered, utilizing immunomodulatory and proteasome inhibitors that have not been used during induction treatment. Furthermore, venetoclax may represent an option in cohorts with t(11;14) [18].

## Conclusions

PCL is one of the rarest monoclonal gammopathies, known for its variable presentations and aggressive natural course. It can arise as a primary disorder ‘de-novo’ or transform from previously diagnosed multiple myeloma. Although representing a subset of plasma cell dyscrasia, PCL is a distinct entity from multiple myeloma with different therapeutic modalities. Despite the introduction of novel agents, the prognosis of PCL is inferior compared to other plasma cell dyscrasias. Further research is needed to better establish targeted therapies with an aim to improve survival.
